# Correction: The Effect of Increasing Water Temperatures on *Schistosoma mansoni* Transmission and *Biomphalaria pfeifferi* Population Dynamics: An Agent-Based Modelling Study

**DOI:** 10.1371/journal.pone.0105917

**Published:** 2014-08-11

**Authors:** The PLOS ONE Staff

## Notice of Republication

This article was republished on July 29, 2014, to correct the PDF, which used an incorrect version of the manuscript. The publisher apologizes for the error. Please download this article again to view the correct version. The originally published, uncorrected article and the republished, corrected article are provided here for reference.

## Supporting Information

File S1 Originally published, uncorrected article.
(PDF)

File S2 Republished, corrected article.
(PDF)
