# Supplementary material for: The Effect of Increasing Water Temperatures on Schistosoma mansoni Transmission and Biomphalaria pfeifferi Population Dynamics: An Agent-Based Modelling Study
Source: PLoS One. 2014 Jul 2;9(7):e101462. doi: 10.1371/journal.pone.0101462 (PMC4079709; doi:10.1371/journal.pone.0101462)
Supplement: Model Description S1 — Additional information on the model structure and parameterisation. (DOCX) [file pone.0101462.s001.docx]

**Model description S1**

**Temperature dependent rates**

The model contains a number of temperature dependent rates. In general, only a small number of data points were available for estimating the relationship between temperature and the rate in each case. To calculate the rates used in the model at temperatures between the data points, linear, quadratic, exponential, Weibull, Lactin[[1](#_ENREF_1)], Gompertz or piecewise equations were fitted to the data points using a least squares method. The choice of equation to use in each case was decided by a combination of looking at plots of the data, and knowledge of which relationships were biological plausible. Table S1 gives details of all of the rates used in the model.

Unless stated otherwise, all data used in the development of the model come from studies of *Biomphalaria pfeifferi* snails and *Schistosoma mansoni*.

**Snails**

Snails in the model have three life stages: egg, juvenile and adult. Adults and juveniles can be uninfected, prepatent (infected but not yet producing cercariae) or infectious (infected and producing cercariae). Adult snails are able to produce eggs.

**Juvenile development**

Data on the number of days between hatching and the start of egg laying at known temperatures for *B. pfeifferi* were available from a number of different laboratory studies[[2-8](#_ENREF_2)] (figure S1a), and from one field study at a mean water temperature of 25.6°C[[9](#_ENREF_9)]. They were translated into heat units gained per hour, with 100 units necessary to start egg laying. The concept of heat units is used to determine the rate at which snails move between life-stages. The concept is similar to obtaining sufficient energy or credit to achieve a specific goal. In this system, the goal is movement to the next life-stage. A ‘heat unit’ approach allows the degree of development of the snail to be tracked in the model if fluctuating temperatures are simulated, and, unlike a degree-day approach, does not make an assumption of linearity[[10](#_ENREF_10)]. Data were available from one of the laboratory studies (de Kock and van Eeden[[7](#_ENREF_7)]) at temperatures of 17°C, 20°C, 23°C, 26°C, 29°C and 32°C. The data from this single study were fitted with a Lactin equation. The other laboratory studies each gave data from only one to three different water temperatures each, and showed variation between studies. All found much slower rates of development than de Kock and van Eeden however, as did the field study. The fitted Lactin model rates were therefore reduced by a factor of 0.54, which caused them to pass through the single data point available from a field study, and this scaled Lactin model was used to simulate juvenile snail development in the model (figure S1a). The fitted equation met the x-axis at 6.1°C and 33.6°C and therefore no development was assumed to occur below and above these temperatures respectively.

A field study which compared the number of snails in a generation with the temperatures during the development period of its parents’ generation found that fecundity appeared to decrease above a critical threshold of mean weekly levels of 120-179 degree hours above 27°C, equivalent to 17-26 degree hours above 27°C a day[[11](#_ENREF_11)]. This was investigated further with a laboratory study where developing snails were kept in tanks at diurnally fluctuating temperatures. The study found that both gonad development and egg production were impaired in snails exposed to above 39 degree hours above 27°C a day[[12](#_ENREF_12)], and snails kept at the highest temperature regime of 75 degree hours above 27°C a day produced very few eggs. The author suggests that the reason for the different threshold found in the two studies is that in the field there was much variation between days and weeks in the number of degree hours above 27°C a day. This suggests that short periods of very high temperatures are more damaging to snail development than longer periods of more moderate high temperatures.

In the model, during each juvenile snail’s development period a record is kept of the total number degree hours spent above 27°C, above a threshold of 39 degree hours above 27°C a day. For example, if one day contains 52 degree hours above 27°C then the snail’s total number of degree hours above 27°C will be increased by 52 - 39 = 13 degree hours. The snail’s total number of degree hours above 27°C will not be increased on days where there are ≤39 degree hours above 27°C. When the snail has accumulated sufficient heat units to start producing eggs, its high temperature egg production weight is calculated. If the number of degree hours above 27°C was ≤39 degree hours every day during its development, the weight is set equal to one. If the number of degree hours above 27°C was >39 degree hours on any day, then the value of the weight is determined from an exponential line fitted to the data described above (figure S1b).

**
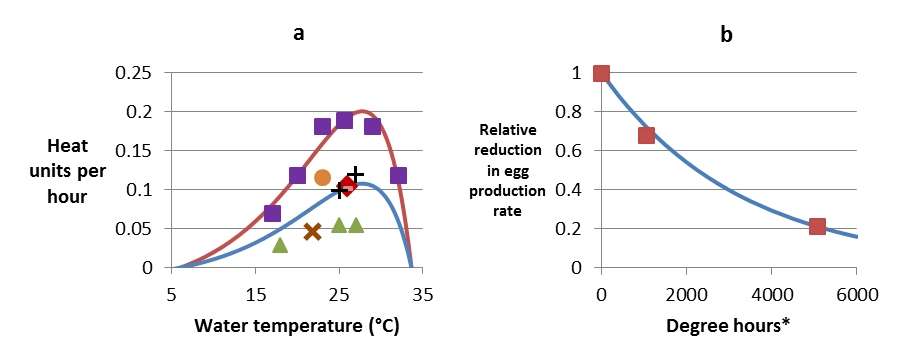
**

**Figure S1. Juvenile development rate graphs.** a) Rate of juvenile heat unit gain per hour by water temperature. 100 heat units are necessary to complete development and start egg laying. The green triangles[[2](#_ENREF_2)], brown cross[[3](#_ENREF_3)], black crosses[[4](#_ENREF_4)], orange circle[[8](#_ENREF_8)], and purple squares[[7](#_ENREF_7)] show empirical data from laboratory studies. The red diamond shows empirical data from a field study[[9](#_ENREF_9)]. The red line is a Lactin model fitted to the de Kock and van Eeden (1981)[[7](#_ENREF_7)] data. The blue line is the Lactin model scaled to pass through the field data point, and the relationship used in the model. b) Effect of high temperatures during juvenile development on the adult egg production rate. Red squares show data[[12](#_ENREF_12)] and the blue lines shows the relationship used in the model. *Cumulative number of degree hours above 27°C, above a threshold of 39 degree hours above 27°C per day

**Egg production**

De Kock and van Eeden recorded the mean number of eggs produced per snail every two weeks for snails kept at constant temperatures of 17°C, 20°C, 23°C, 26°C, 29°C and 32°C in a laboratory[[7](#_ENREF_7)]. These were converted into hourly rates and fitted with a Lactin model (Figure S2a. A field study suggests that egg production rates in a more natural environment may be much lower however[[9](#_ENREF_9)]. The highest rate of egg production found in the field study was only 0.22 times the rate predicted by the Lactin model for the same temperature. The simulated maximum egg production rate by water temperature was therefore taken to be equal to the value given by the Lactin model times 0.22 (figure S2b). No egg production occurs in the model below 13.8°C and above 32.1°C where the model crosses the x-axis.

In laboratory studies the vast majority of snail eggs kept in suitable conditions successfully hatch and survive the first few days after hatching[[6](#_ENREF_6)]. This is not the case in more natural conditions. At one field site where water temperatures ranged from 17-22°C it was estimated that only 2.7-8.5% of eggs laid hatched and survived the period immediately following hatching[[13](#_ENREF_13)]. Simulating the eggs that do not survive would have no effect on the model other than to slow it down, and therefore this initial high mortality is simulated in the model by reducing birth rates to 10% of the rate they would otherwise take. The number of eggs that successfully hatch is then reduced further by an additional temperature dependent egg mortality, which is described in the section on egg hatching.

Adult *B. pfeifferi* kept at 25°C and infected with *S. mansoni* ceased to produce eggs when they were around halfway through their pre-patent period[[14](#_ENREF_14)]. Simulated prepatent snails therefore stopped producing eggs after they had accumulated 50% of the heat units necessary to become infectious (see the section below on parasite development within snails for an explanation of heat units).

Overall egg production in the model is therefore calculated for each snail per hour as follows. Firstly, snails which have not accumulated sufficient heat units to become adults and snails which are infectious or more than halfway through their prepatent period do not produce eggs. Secondly, the maximum possible rate of egg production that hour, given the water temperature, is calculated. The rate is then adjusted for snail density, as described in the density dependence section below and for high temperatures during development, as described above. High mortality before and during hatching is then accounted for by multiplying the rate by 0.1. Finally, a floating point number between 0 and 1 is chosen at random for that snail. If the number is less than the final egg production rate, the snail lays an egg. This process is repeated for each snail, and repeated for all snails every hour.


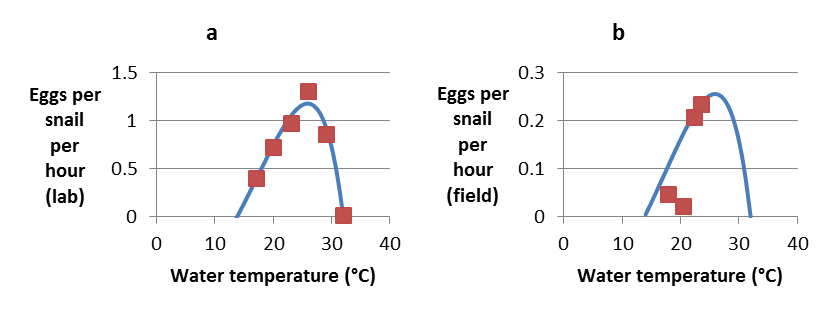


**Figure S2. Egg production graphs.** a) Egg production rate per snail per hour in a laboratory. The red squares give data from a laboratory study[[7](#_ENREF_7)] and the blue line gives a Lactin model fitted to the data. b) Egg production rate per snail per hour in the field. The red squares give data from a field study[[9](#_ENREF_9)] and the blue line gives a scaled version of the Lactin model shown in a. This scaled Lactin model is used in the dynamical model.

**Egg development**

De Kock and van Eeden record the number of days until egg hatching for eggs *B. pfeifferi* kept at constant temperatures of 17°C, 20°C, 23°C, 26°C, 29°C and 32°C in a laboratory[[7](#_ENREF_7)]. Their findings were consistent with the number of days until hatching recorded for snail eggs kept in cages in two streams during two seasons in Kenya, with mean water temperatures of 19.7°C, 21.5°C, 24.6°C and 25.6°C[[9](#_ENREF_9)]. The laboratory data were converted into heat unit gain per hour, with 100 heat units necessary for hatching (Figure S3a). A linear line was fitted through these points and was used to simulate egg development rates in the model. No egg development is assumed to occur below 5.9°C in the model where the fitted line meets the x-axis.

In De Kock and van Eeden’s experiments, 91-96% of snail eggs hatched between 17°C and 29°C, and 75% hatched at 32°C[[7](#_ENREF_7)]. The proportions of egg that hatched and the number of days until hatching were converted into mortality rates per hour for each temperature (Figure S3b). Below 29°C, a constant mortality rate was modelled. Above 29°C, the simulated
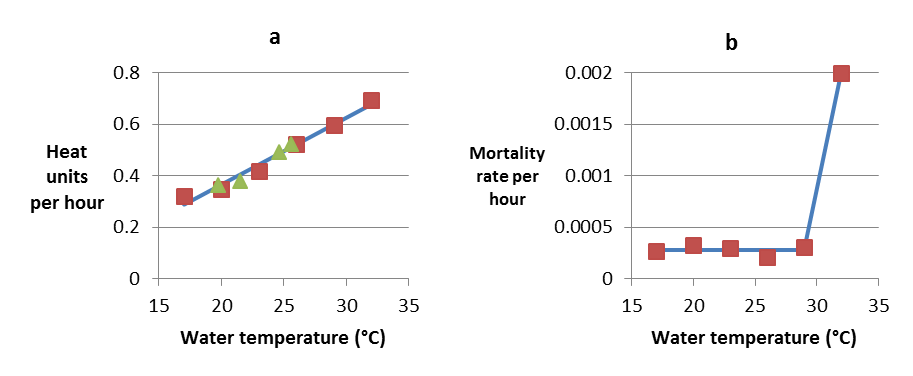
 egg mortality rate increases linearly with temperature.

**Figure S3. Egg development graphs.** a) Rate of heat unit gain per hour. 100 heat units are needed for eggs to hatch. The red squares show experimental data[[7](#_ENREF_7)], the green triangles show field data[[9](#_ENREF_9)], and the blue lines shows the relationship used in the model. b) Egg mortality rate per hour. The red squares show experimental data[[7](#_ENREF_7)] and the blue line shows the relationship used in the model.

**Mortality**

Although laboratory studies can show sharp increases in mortality at around 10 months of age[[3](#_ENREF_3)], snails can be found in the field with sizes corresponding to ages of around 19 months[[13](#_ENREF_13)]. A longitudinal field study at a site in DR Congo where water temperatures varied between 17-22°C (mean 19°C) suggested that, after hatching, the mortality rate of *B. pfeifferi* was approximately constant at 0.00037/hour in 1982 and 0.00021/hour in 1984[[13](#_ENREF_13)]. The mortality rate at 19°C in the model was therefore taken to be the average of these two values.

Data on effect of temperature on *B. pfeifferi* mortality is sparse. Woolhouse *et al.* estimated mortality rates in a field study at a range of mean weekly water temperatures between 13-25°C using a mark-recapture method[[15](#_ENREF_15)]. The best linear fit trend suggested that mortality increases slightly with increasing temperature within this range, but the standard errors of the data points were large and the results were consistent with a range of different relationships. De Kock and Van Eeden recorded weekly survival rates for snails kept in a laboratory at 17°C, 20°C, 23°C, 26°C, 29°C and 32°C[[7](#_ENREF_7)]. A linear line was fitted through median survival times and used to estimate the mortality rate at 19°C. The mortality rates at each temperature relative to the mortality rate at 19°C were then calculated. A linear line was fitted through these relative mortality rates and this, in combination with the field mortality rate at 19°C, was used to calculate the mortality rate at temperatures between 13°C (the lower end of the temperature range for which Woolhouse *et al*. estimated field mortality rates[[15](#_ENREF_15)]) and 32°C (figure S4a).

Joubert *et al* measured *B. pfeifferi* mortality at very low temperatures (0°C, 2°C, 4°C and 6°C) in a laboratory study[[16](#_ENREF_16)] (figure S4b). Mortality rates increased sharply with decreasing temperature, with median survival rates for the snails varying between 25 hours at 0° and 75 hours at 6°C. A quadratic equation was fitted between the four low temperature data points and the simulated mortality rate at 13°C and this was used to calculate mortality rates below 13°C in the model.

A second study investigated *B. pfeifferi* mortality in a laboratory at high temperatures (34°C, 36°C, 38°C and 40°C)[[17](#_ENREF_17)] (Figure S4b). Median survival at these temperatures ranged from around 3 hours at 40°C to 8 days at 34°C. An exponential equation was fitted between the four high temperature data points and the simulated mortality rate at 32°C and this was used to calculate mortality rates above 32°C in the model.

Foster[[18](#_ENREF_18)] compared mortality rates in shedding and non-shedding *B. pfeifferi* kept at four temperatures between 23-28°C (Figure S4c). Mortality rates were found to be 2.1-6.6 times higher in shedding snails, with the ratio increasing with increasing temperature. It was assumed that the ratio was equal to one below 11.2°C, the estimated temperature below which snails do not produce cercariae. The best fit exponential trend line was fitted between the five points, and this was used to model increased mortality in infectious snails compared to uninfected snails within the temperature range above 11.2°C. Below 11.2°C the modelled ratio was equal to one.

Prepatent *B. glabrata* show no increase in mortality compared to uninfected controls[[19](#_ENREF_19)]. No increase in mortality rates was therefore simulated for prepatent snails.


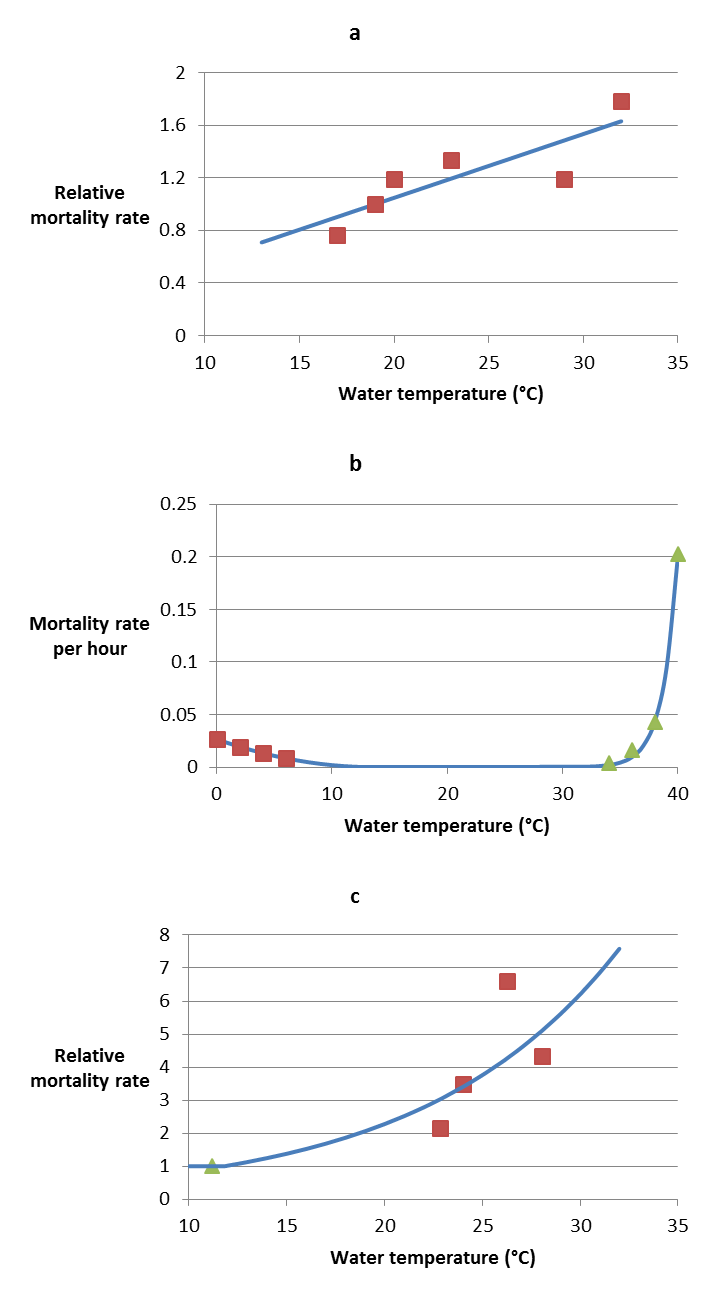


**Figure S4. Snail mortality rates.** a) Mortality rates relative to the mortality rate at 19°C. The blue line shows the relationship used in the model. Red squares show data from de Kock and van Eeden[[7](#_ENREF_7)]. b) Snail morality rates per hour at extreme temperatures. The blue line shows the relationship used in the model. The red squares[[16](#_ENREF_16)] and green triangles[[17](#_ENREF_17)] show experimental data. c) Mortality rates in infectious snails relative to mortality rates in uninfected snails. The blue line shows the relationship used in the model. The red squares show experimental data[[18](#_ENREF_18)]. The green triangle shows the estimated temperature below which snails do not produce cercariae.

**Snail density dependence**

In laboratory conditions, a high density of snails did not affect the time to first or maximum egg laying, or snail mortality rates[[3](#_ENREF_3)]. It did however reduce the number of fertile eggs laid by each snail each week. Field studies support the idea that unfavourable conditions have a greater effect on egg production than snail mortality. A longitudinal study of a snail population in a stream, where water temperatures were favourable for snails all year round, found a 12 fold variation in estimated egg production rates per snail over the course of the study, but little variation in life expectancy after hatching[[13](#_ENREF_13)].

In the model, it is assumed that the environment can support 300 snails with no negative effect on egg production. Above this number, the rate of egg production drops following a Gompertz distribution (figure S5a). At population numbers of more than 600 snails, snail mortality rates also increase (figure S5b).


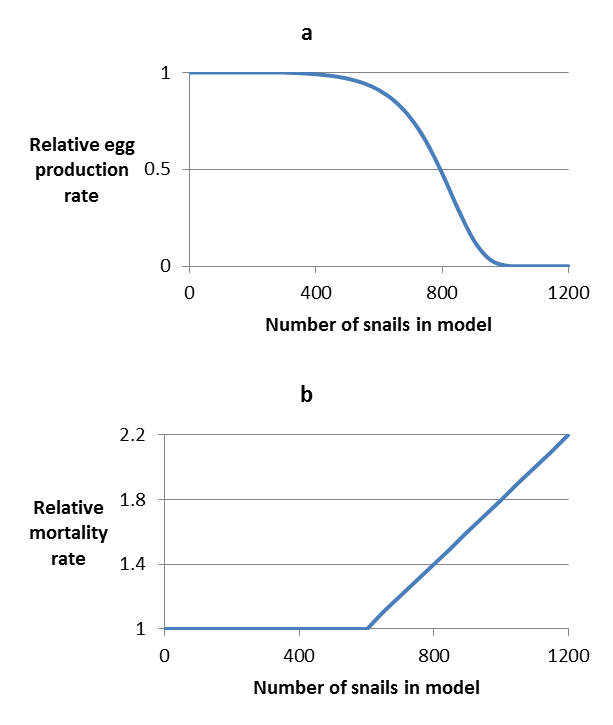


**Figure S5. Effect of large numbers of simulated snails on simulated egg production and mortality rates.** a) Simulated drop in egg production rates at high snail numbers. b) Simulated increase in snail mortality rates at high snail numbers.

**Parasite**

**Parasite development within the snail**

Once a snail becomes infected in the model it starts to accumulate heat units at a temperature dependent rate. This represents parasite development within the snail. When a sufficient number of heat units have been accumulated the snail becomes infectious.

Foster measured the mean time between infection and the start of shedding for *S. mansoni* in *B. pfeifferi* kept at eight temperatures between 18°C and 32°C[[18](#_ENREF_18)] (Figure S6). Parasite development took only 17.5 days at 32°C, compared to 57 days at 18°C, and the data showed a clear linear trend. This was converted into a heat unit accumulation rate, with 100 heat units needed for the snail to become infectious, and was used to simulate parasite development with the temperature range 17-32°C.

Pflüger measured prepatency times at different fluctuating temperature regimes for *S. mansoni* in *B. glabrata*[[20](#_ENREF_20)]. He found that the assumption of a linear relationship between temperature and development rate does not hold for temperature regimes incorporating temperatures outside the range of approximately 16-32°C. Outside this range, development periods were shorter and longer than would be expected at low temperatures and high temperatures respectively. A non-linear relationship was therefore modelled for temperatures less than 17°C and greater than 32°C. For temperatures less than 17°C, the relationship was estimated. For temperatures greater than 32°C, a quadratic equation was fitted through five data points from experiments with *B. glabrata* at 32°C to 35°C[[21](#_ENREF_21)], and scaled to pass through the estimated rate of development in *B. pfeifferi* at 32°C.


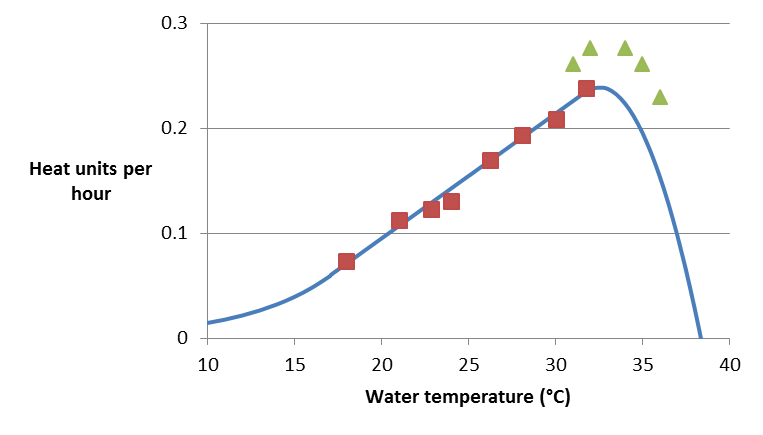


**Figure S6. Parasite development within the snail.** Rate of heat unit gain per hour, with 100 heat units needed for the snail to become infectious. The blue line shows the relationship used in the model. The red squares show data from Foster[[18](#_ENREF_18)]. Green squares show data for *B. glabrata*[[21](#_ENREF_21)]

**Miracidia**

Miracidia are born into the model at a constant rate.

In the real world, miracidia are born with an energy reserve which depletes over time[[22](#_ENREF_22)]. As their energy reserves get lower, their mortality increases and the probability of them successfully infecting a snail decreases[[23](#_ENREF_23)]. The rate at which their energy reserves are depleted is temperature dependent[[22](#_ENREF_22)].

Anderson *et al.* measured the survival over time of *S. mansoni* miracidia kept at eight constant temperatures between 5°C and 40°C[[23](#_ENREF_23)]. Median life expectancy was highest at 15°C. Gompertz functions were fitted to the survival curves at each temperature ≥15°C. Below a constant temperature of 10-15°C, miracidia mortality rates do not increase with age, suggesting that the miracidia may be inactive below this temperature. In the model, when the water temperature is ≥15°C biological age is used instead of chronological age to determine mortality rates by age. This allows the rate of aging to be calculated for snails living in fluctuating temperature regimes. Biological age gain each hour at water temperatures ≥15°C was calculated as a function of current age and water temperature, with biological age gain equal to chronological age gain at 25°C (figure S7a). Mortality rates each hour at each biological age were taken to be equal to the mortality rates at 25°C at the same chronological age, adjusted for the amount of biological age the miracidia had gained over the preceding hour (figure S7b). Above a biological age of 10.6 hours all miracidia die. Below 15°C, biological age does not increase with time, and a temperature dependent, age independent mortality rate is modelled (figure S7c). An additional, constant mortality rate each hour can also be modelled, representing the washing away of miracidia in moving water or mortality due to predation.

Anderson *et al.* also measured the rates of infection of a *B. glabrata* snail by miracidia of different ages kept at five temperatures between 15°C and 35°C[[23](#_ENREF_23)]. The rate of infection at age zero was highest at 25°C, and lower at higher and lower temperatures. The rate of infection decreased with miracidia age at all temperatures, but more rapidly at higher temperatures. In the model, the decline in infection rate with biological age was taken to be equal to the decline in infection rate with chronological age at 25°C, which was estimated using a Weibull model fitted to the empirical infection rates (figure S7d). No infection occurs in the model at water temperatures <15°C.

Each hour, every miracidium in the model has the chance to infect a snail. The probability of infection is dependent on the biological age of the miracidium, the water temperature, and the number of snails in the model. The effect of biological age on infection probability is described above. Exponential lines were fitted to the rates of infection for a miracidia of age zero at 15°C, 20°C and 25°C, and at 25°C, 30°C and 35°C and these relationships were used to model the relative probabilities of infection at different water temperatures (figure S7e). The probability of infection increases with the number of snails in the model according to a cumulative distribution function, however the relationship between the number of snails in the model and infection probability is approximately linear within the range of the number of snails typically found in the model at any time (figure S7f).

When it is determined that a miracidium should infect a snail, the snail is chosen at random from all snails in the model. If the snail is uninfected, the snail becomes prepatent and the miracidium dies. If the snail is already prepatent or infectious then the miracidium dies, but there is no change to the snail.

**Figure S7. Miracidium aging, mortality and infection rates.** a) The simulated increase in biological age with chronological age at different water temperatures. b) The simulated relationship between biological age and miracidia mortality rate per hour, for water temperatures ≥15°C. c) The simulated relationship between water temperature and miracidia mortality rate per hour, for water temperatures <15°C. d) The simulated relationship between biological age and the relative miracidia infection rate. e) The simulated relationship between water temperature and the relative miracidia infection rate. f) The simulated relationship between the number of snails in the model and the relative miracidia infection rate.

**Cercariae**

In the model, cercariae are produced by infected snails at a rate which is dependent on temperature and the time of day.

Kazibwe *et al.* counted the number of cercariae emerging from *B. stanleyi* snails hourly between 9.00 and 17.00[[24](#_ENREF_24)]. The snails were shed under natural light. Cercariae emergence was highest at 13.00, and dropped sharply either side of 12.00-14.00. Cercariae production rates relative to the peak production rate at 13.00 were calculated. A quadratic equation was fitted through these relative rates and this was used to determine the time of day dependence of the number of cercariae produced per infected snail each hour in the model (figure S8a). No cercariae production occurs in the model between 17.00 and 9.00.

Fried *et al*[[25](#_ENREF_25)] counted the number of cercariae emerging each hour from shedding *B. glabrata* kept at 12°C, 25°C and 35°C. Cercariae emergence rates increased greatly with increasing temperature, from an average of 21 cercariae per snail at 12°C to 350 cercariae per snail at 35°C. A linear equation was fitted through the points and, after adjusting for time of day, used to calculate the number of cercariae produced per infectious snail per hour in the model (figure S8b). No cercariae production occurs in the model below 11.2°C where the line meets the x-axis.

Lawson and Wilson measured the survival by age of cercariae kept at six constant temperatures between 15-40°C[[26](#_ENREF_26)]. Gompertz functions were fitted to the survival curves at each temperature. Median survival was 31 hours at 15°C and decreased with increasing temperature to only 3 hours at 40°C. Aging and mortality rates of cercariae were simulated in the model in a similar way to the aging and mortality rates of miracidia. Biological age gain was equal to chronological age gain at 25°C (figure S8c), and mortality rates each hour at each biological age were equal to the mortality rates at 25°C at the same chronological age, adjusted for the amount of biological age the miracidia had gained over the preceding hour (figure S8d). As for miracidia, an additional, constant mortality rate can be included in the model, representing the washing away of cercariae in moving water or mortality due to predation.

Two indicators of human infection risk can be outputted by the model. The first is simply the number of cercariae alive in the model at any point in time. A proportion of cercariae that successfully penetrate a potential host’s skin will die without developing into an adult worm however. Ghandour *et al* measured the proportion of cercariae that died during the penetration of mouse skin for 2, 4, 6, 8, 10, 18 and 24 hour old cercariae, kept at 25-27°C[[27](#_ENREF_27)]. The proportion of cercariae that were *not* found dead in the mice’s skin declined exponentially over time from 71% at two hours to 16% at 24 hours. The second measure of human infection risk outputted by the model is therefore calculated as the number of cercariae in the model, adjusted by their biological age dependent probability that they would die during skin penetration (figure S8e).

**Figure S8. Cercaria production, aging, mortality and infection rates.** a) The relative production rate of cercariae by time of day. The blue squares show experimental data[[24](#_ENREF_24)] and the red line shows the modelled relationship b) Maximum rate of cercaria production per hour by water temperature. The blue squares show experimental data[[25](#_ENREF_25)] and the red line shows the modelled relationship. c) The simulated increase in biological age with chronological age at different water temperatures. d) The simulated relationship between biological age and cercariae mortality rate per hour. e) The simulated relationship between cercariae biological age and infection probability. The blue squares show experimental data[[27](#_ENREF_27)] and the red line shows the modelled relationship.

| **Rate (per hour) [varies with]** | **Source(s)** | **Range of data** | **Fitted model** | **Equation/ Parameters** | **Behaviour outside range of data** |
| --- | --- | --- | --- | --- | --- |
| Juvenile heat unit gain* [water temperature (*T_w_*)] | De Kock and van Eeden[[7](#_ENREF_7)] and Kariuki[[9](#_ENREF_9)] | 17-32°C (laboratory) and 25.6°C (field) | Lactin | 0.54 * Lactin model with parameters:  ρ = 0.161  λ = -0.032  Δ = 6.20  T_max_ = 34.0°C | Line extended to 6.1°C and 33.6°C where the model meets the x-axis. No heat unit gain occurs outside this range. |
| Relative egg production rate [Cumulative number of degree hours above 27°C, above a threshold of 39 degree hours above 27°C per day, during a juvenile snail’s development period *(d)*] | Appleton[[12](#_ENREF_12)] | 0-5065 degree hours above 27°C, above a threshold of 39 degree hours above 27°C per day | Exponential | e ^ (-0.00031 * *d*) | Line extended to higher values of *d* |
| Egg production rate [water temperature (*T_w_*)] | De Kock and van Eeden[[7](#_ENREF_7)] and Kariuki[[9](#_ENREF_9)] | 17-32°C (laboratory) and 17.9-23.5°C (field) | Lactin | 0.1 * 0.22 * Lactin model with parameters:  ρ = 0.105  λ = -1.687  Δ = 7.72  T_max_ = 34.5°C | Line extended to 13.8°C and 32.1°C where the model meets the x-axis. No egg production occurs outside this range. |
| Egg heat unit gain*  [water temperature (*T_w_*)] | De Kock and van Eeden[[7](#_ENREF_7)] and Kariuki[[9](#_ENREF_9)] | 17-32°C (laboratory) and 19.7-25.6°C (field) | Linear | 0.0259 * *T_w_* - 0.152 | Line extended in both directions. No egg heat unit gain occurs below 5.9°C where the line meets the x-axis |
| Egg mortality rate  [water temperature (*T_w_*)] De Kock and van Eeden[[7](#_ENREF_7)] | De Kock and van Eeden[[7](#_ENREF_7)] | 17-32°C | Piecewise linear | If *T_w_* < 29°C: 0.000283  If *T_w_* ≥ 29°C:  0.000564 * *T_w_* - 0.0161 | Lines extended to higher and lower temperatures |
| Mortality rate at 19°C | Loreau and Baluku[[13](#_ENREF_13)] | - | - | 0.000285 | - |
| Relative mortality rate between 13-32°C (relative to rate at 19°C)  [water temperature (*T_w_*)] | De Kock and van Eeden[[7](#_ENREF_7)] | 17-32°C | Linear | 0.0485 * *T_w_* + 0.0776 | See rows below |
| Mortality rate below 13°C  [water temperature (*T_w_*)] | Joubert *et al[*[*16*](#_ENREF_16)*]* | 0-6°C | Quadratic | 0.000141 * $T_{w}^{2}$ - 0.00388 * *T_w_* + 0.0268 | Line extended below 0°C |
| Mortality rate above 32°C  [water temperature (*T_w_*)] | Joubert *et al[*[*17*](#_ENREF_17)*]* | 34-40°C | Exponential | 1.30 * 10^-14^ * e ^ (0.76 * *T_w_*) | Line extended above 40°C |
| Relative increase in mortality in infectious snails [water temperature (*T_w_*)] | Foster[[18](#_ENREF_18)] | 23-28°C | Exponential | 0.3073 * e ^ (0.1002 * *T_w_*) | Line extended backwards to 11.2°C where it takes the value of 1. Below 11.2°C, the relative increase in mortality is taken to be equal to 1. Line extended forwards above 28°C. |
| Relative increase in mortality [number of snails in model *N_s_*] | - | - | Linear | If *N_s_* < 600: 1  If *N_s_* ≥ 600:  (*N_s_* – 600) / 500 + 1 | Lines used for any number of snails in model |
| Relative egg production rate [number of snails in model *N_s_*] | Loreau and Baluku[[3](#_ENREF_3)] | Data from tanks with 8.6 snails/l and 85.7 snails/l only | Gompertz | If *N_s_* < 300: 1  If *N_s_* ≥ 300:  e ^ (0.005 * (1 – e ^ ((*N_s_* -300)/100))) | Lines used for any number of snails in model |
| Parasite heat unit gain within snail* [water temperature (*T_w_*)] | Foster[[18](#_ENREF_18)] and Pflüger[[20](#_ENREF_20)] | 18-32°C (constant temperature) 12-39°C (as part of a fluctuating regime) | Piecewise linear, quadratic and exponential | If *T_w_* < 17°C:  (0.005 * e ^ ((*T_w_* - 4.7) / 5))  If *T_w_* ≥ 17°C and *T_w_* < 32°C:  0.0119 * *T_w_* - 0.143  If *T_w_* ≥ 32°C: 0.87 * (-0.0080 * *T_w_^2^* + 0.52 * *T_w_* - 8.18) | Line extended in both directions. No parasite heat unit gain occurs above 38.4°C where the line meets the x-axis |
| Miracidium biological age gain [water temperature (*T_w_*)] | Anderson *et al[*[*23*](#_ENREF_23)*]* | 5-40°C | Piecewise linear | If *T_w_* < 15°C: 0  If *T_w_* ≥ 15°C:  0.0530 * *T_w_* - 0.349 | No biological age gain occurs below 15°C. The line is extended forwards above 40°C |
| Miracidium mortality rate if *T_w_* ≥ 15°C [miracidium biological age (hours) (*M_a_*)] | Anderson *et al[*[*23*](#_ENREF_23)*]* | Miracidium birth to 100% mortality of cohort | Exponential | 1 - (1 - (0.0083 * e ^ ((-0.35 + 0.032 * 25) * *M_a_*))) | Line used to determine mortality rate at all biological ages |
| Miracidium mortality rate if *T_w_* < 15°C [water temperature (*T_w_*)] | Anderson *et al[*[*23*](#_ENREF_23)*]* | Miracidium birth to 100% mortality of cohort | Linear | -0.0192 * *T_w_* + 0.2911 | Line used to determine mortality rate for all water temperatures < 15°C |
| Relative miracidium infection rate [miracidium biological age (hours) (*M_a_*)] | Anderson *et al[*[*23*](#_ENREF_23)*]* | Miracidium birth to 100% mortality of cohort | Weibull | (0.196 * e ^ ((-2.18 * 10^-6^ / D$6) * *M_a_* ^ 8.33)) / 0.196 | Line used to determine relative infection rate at all biological ages |
| Relative miracidium infection rate [water temperature (*T_w_*)] | Anderson *et al[*[*23*](#_ENREF_23)*]* | 15-35°C | Piecewise exponential | If *T_w_* < 15°C: 0  If 15°C ≤ *T_w_* ≤ 25°C:  0.135 * e ^ (0.0572 * *T_w_*)  If *T_w_* > 25°C:  2.28 * e ^ (-0.0544 * *T_w_*) | Lines extended to higher and lower temperatures |
| Relative miracidium infection rate [number of snails in model *N_s_* | - | - | 1 - exponential | 1 – e ^ (-*N_s_* / 3000) | Line used for any number of snails in model |
| Relative cercaria production rate [time of day (hours since midnight *h*)] | Kazibwe[[24](#_ENREF_24)] | 9.00-17.00 | Quadratic | -0.0578 * *h*^2^ + 1.501 * *h* - 8.84 | No cercariae production occurs between 17.00 and 9.00 |
| Cercaria production rate [water temperature (*T_w_*)] | Fried *et al[*[*25*](#_ENREF_25)*]* | 12-35°C | Linear | 0.02 * (14.2 * *T_w_* - 159.2) | Line extended in both directions. No cercariae production occurs below 11.2°C where the line meets the x-axis |
| Cercaria biological age gain (hours) [water temperature (*T_w_*)] | Lawson and Wilson[[26](#_ENREF_26)] | 15-40°C | Exponential | 0.199 * e ^ (0.069 * *T_w_*) | Lines extended to higher and lower temperatures |
| Cercaria mortality rate [cercaria biological age (hours) (*C_a_*)] | Lawson and Wilson[[26](#_ENREF_26)] | Cercaria birth to 100% mortality of cohort | Gompertz | 1 – (1 – e ^ (-e ^ (1.75 – 0.056 * *C_a_*))) ^ (*cercaria age gain*) | Line used to determine mortality rate at all biological ages |
| Relative cercaria infection rate [cercaria biological age (hours) (*C_a_*)] | Ghandour *et al[*[*27*](#_ENREF_27)*]* | 2-24 hours | Exponential | 0.829 * e ^ (-0.0675 * *C_a_*) | Line used to determine relative infection rate at all biological ages |

**Table S1. Model rates** *Number of heat units necessary to complete stage set to 100

**1. Lactin DJ, Holliday NJ, Johnson DL, Craigen R (1995) Improved Rate Model of Temperature-Dependent Development by Arthropods. Environmental Entomology 24: 68-75.**

**2. Shiff CJ, Garnett B (1963) The influence of temperature on the intrinsic rate of natural increase of the freshwater snail Biomphalaria pfeifferi (Krauss). Archiv für Hydrobiologie 62: 429–438.**

**3. Loreau M, Baluku B (1987) Growth and demography of populations of *Biomphalaria pfeifferi* (gastropoda, planorbidae) in the laboratory. Journal of Molluscan Studies 53: 171-177.**

**4. Appleton CC (1977) The influence of above-optimal constant temperatures on South African Biomphalaria pfeifferi (Krauss) (Mollusca: Planorbidae). Trans R Soc Trop Med Hyg 71: 140-143.**

**5. Nduku WK, Harrison AD (1976) Calcium as a limiting factor in the biology of Biomphalaria pfeifferi (Krauss), (Gastropoda: Planorbidae). Hydrobiologia 49: 143-170.**

**6. Sturrock RF (1966) The influence of temperature on the biology of Biomphalaria pfeifferi (Krauss), an intermediate host of Schistosoma mansoni. Ann Trop Med Parasitol 60: 100-105.**

**7. De Kock KN, Van Eeden JA, Slaknavorsingseenheid PUfCHE, Unit SAMRCSR (1981) Life Table Studies on Freshwater Snails: The effect of constant temperature on the population dynamics of Biomphalaria pfeifferi (Krauss): Potchefstroom University for CHE Publications.**

**8. de Kock KN, Van Eeden JA (1986) Effect of programmed circadian temperature fluctuations on population dynamics of Biomphalaria pfeifferi(Krauss). South African Journal of Zoology 21: 28-32.**

**9. Kariuki C (1994) Comparative ecological studies on transmission of Schistosoma mansni and population dynamics, taxonomy and molluscicide sensitivity of Biomphalaria pfeifferi, intermediate host of S. mansoni , in two areas of Machakos district, Kenya: University of Copenhagen.**

**10. McCreesh N, Booth M (2013) Challenges in predicting the effects of climate change on Schistosoma mansoni and Schistosoma haematobium transmission potential. Trends in Parasitology 29: 548-555.**

**11. Appleton CC (1977) The influence of temperature on the life-cycle and distribution of Biomphalaria pfeifferi (Krauss, 1948) in South-Eastern Africa. International Journal for Parasitology 7: 335-345.**

**12. Appleton CC, Eriksson IM (1984) The influence of fluctuating above-optimal temperature regimes on the fecundity of Biomphalaria pfeifferi (Mollusca: Planorbidae). Transactions of the Royal Society of Tropical Medicine and Hygiene 78: 49-54.**

**13. Loreau M, Baluku B (1987) Popoulation dynamics of the freshwater snail Biomphalaria pfeifferi in Eastern Zaire. Journal of Molluscan Studies 53: 249-265.**

**14. Meuleman EA (1971) Host-Parasite Interrelationships Between the Freshwater Pulmonate Biomphalaria Pfeifferi and the Trematode Schistosoma Mansoni. Netherlands Journal of Zoology 22: 355-427.**

**15. Woolhouse MEJ (1992) Population Biology of the Freshwater Snail Biomphalaria pfeifferi in the Zimbabwe Highveld. Journal of Applied Ecology 29: 687-694.**

**16. Joubert P, Pretorius S, De Kock K, Van Eeden J (1984) The effect of constant low temperatures on the survival of Bulinus africanus(Krauss), Bulinus globosus(Morelet) and Biomphalaria pfeifferi(Krauss). S AFR J ZOOL/S-AFR TYDSKR DIERKD 19: 314-316.**

**17. Joubert P, Pretorius S, De Kock K, Van Eeden J (1986) Survival of Bulinus africanus (Krauss), Bulinus globosus (Morelet) and Biomphalaria pfeifferi (Krauss) at constant high temperatures. S Afr J Zool 21: 85-88.**

**18. Foster R (1964) The Effect of Temperature on the Development of Schistosoma Mansoni Sambon 1907 in the Intermediate Host. J Trop Med Hyg 67: 289-292.**

**19. Minchella DJ, Loverde PT (1981) A cost of increased early reproductive effort in the snail Biomphalaria glabrata. The American Naturalist 118: 876-881.**

**20. Pfluger W (1981) Experimental epidemiology of schistosomiasis. II. Prepatency of Schistosoma mansoni in Biomphalaria glabrata at diurnally fluctuating temperatures. Zeitschrift fur Parasitenkunde (Berlin, Germany) 66: 221-229.**

**21. Pfluger W (1980) Experimental epidemiology of schistosomiasis. I. The prepatent period and cercarial production of Schistosoma mansoni in Biomphalaria snails at various constant temperatures. Z Parasitenkd 63: 159-169.**

**22. MORLEY NJ (2012) Thermodynamics of miracidial survival and metabolism. Parasitology 139: 1640-1651.**

**23. Anderson RM, Mercer JG, Wilson RA, Carter NP (1982) Transmission of Schistosoma mansoni from man to snail: experimental studies of miracidial survival and infectivity in relation to larval age, water temperature, host size and host age. Parasitology 85 (Pt 2): 339-360.**

**24. Kazibwe F, Makanga B, Rubaire-Akiiki C, Ouma J, Kariuki C, et al. (2010) Transmission studies of intestinal schistosomiasis in Lake Albert, Uganda and experimental compatibility of local Biomphalaria spp. Parasitol Int 59: 49-53.**

**25. Fried B, LaTerra R, Kim Y (2002) Emergence of cercariae of Echinostoma caproni and Schistosoma mansoni from Biomphalaria glabrata under different laboratory conditions. J Helminthol 76: 369-371.**

**26. Lawson JR, Wilson RA (1980) The survival of the cercariae of Schistosoma mansoni in relation to water temperature and glycogen utilization. Parasitology 81: 337-348.**

**27. Ghandour AM, Webbe G (1973) A study of the death of Schistosoma mansoni cercariae during penetration of mammalian host skin: the influence of the ages of the cercariae and of the host. Int J Parasitol 3: 789-794.**
