# Supplementary material for: Correction: The Effect of Increasing Water Temperatures on Schistosoma mansoni Transmission and Biomphalaria pfeifferi Population Dynamics: An Agent-Based Modelling Study
Source: PLoS One. 2014 Aug 11;9(8):e105917. doi: 10.1371/journal.pone.0105917 (PMC4128806; doi:10.1371/journal.pone.0105917)
Supplement: File S2 — Republished, corrected article. (PDF) [file pone.0105917.s002.pdf]

The Effect of Increasing Water Temperatures on *Schistosoma mansoni* Transmission and *Biomphalaria pfeifferi* Population Dynamics: An Agent-Based Modelling Study

Nicky McCreesh*, Mark Booth

School of Medicine, Pharmacy and Health, Durham University, Durham, United Kingdom

Abstract

Introduction: There is increasing interest in the control and elimination of schistosomiasis. Little is known, however, about the likely effects of increasing water-body temperatures on transmission.

Methods: We have developed an agent-based model of the temperature-sensitive stages of the *Schistosoma* and intermediate host snail life-cycles, parameterised using data from *S. mansoni* and *Biomphalaria pfeifferi* laboratory and field-based observations. Infection risk is calculated as the number of cercariae in the model, adjusted for their probability of causing infection.

Results: The number of snails in the model is approximately constant between 15–31°C. Outside this range, snail numbers drop sharply, and the snail population cannot survive outside the range 14–32°C. Mean snail generation time decreases with increasing temperature from 176 days at 14°C to 46 days at 26°C. Human infection risk is highest between 16–18°C and 1 pm and 6–10 pm in calm water, and 20–25°C and 12–4 pm in flowing water. Infection risk increases sharply when temperatures increase above the minimum necessary for sustained transmission.

Conclusions: The model suggests that, in areas where *S. mansoni* is already endemic, warming of the water at transmission sites will have differential effects on both snails and parasites depending on abiotic properties of the water-body. Snail generation times will decrease in most areas, meaning that snail populations will recover faster from natural population reductions and from snail-control efforts. We suggest a link between the ecological properties of transmission sites and infection risk which could significantly affect the outcomes of interventions designed to alter water contact behaviour – proposing that such interventions are more likely to reduce infection levels at river locations than lakes, where infection risk remains high for longer. In cooler areas where snails are currently found, increasing temperatures may significantly increase infection risk, potentially leading to new, high-intensity foci of infection.

Citation: McCreesh N, Booth M (2014) The Effect of Increasing Water Temperatures on *Schistosoma mansoni* Transmission and *Biomphalaria pfeifferi* Population Dynamics: An Agent-Based Modelling Study. PLoS ONE 9(7): e101462. doi:10.1371/journal.pone.0101462

Editor: Ulrike Gertrud Munderloh, University of Minnesota, United States of America

Received March 21, 2014; **Accepted** June 6, 2014; **Published** July 2, 2014

Copyright: © 2014 McCreesh, Booth. This is an open-access article distributed under the terms of the Creative Commons Attribution License, which permits unrestricted use, distribution, and reproduction in any medium, provided the original author and source are credited.

Data Availability: The authors confirm that all data underlying the findings are fully available without restriction. All relevant data are within the paper and its Supporting Information files.

Funding: The research leading to these results has received funding from the European Union's Seventh Framework Programme (FP7/2007–2013) under grant agreement no. 266327. The funders had no role in study design, data collection and analysis, decision to publish, or preparation of the manuscript.

Competing Interests: The authors have declared that no competing interests exist.

* Email: Nicky.mccreesh@durham.ac.uk

Introduction

It is increasingly recognised that climate change may have large impacts on many aspects of human health. The first assessment report of the United Nation's Intergovernmental Panel on Climate Change (IPCC), published in 1990 [1], devoted less than four pages to human health. This had increased to an entire chapter in the latest assessment report, published in 2007 [2]. In 2008 the World Health Assembly unanimously adopted a resolution on climate change and health that called on the World Health Organization (WHO) to strengthen its work on climate change and health, and provided a framework for action for both national governments and the WHO [3].

Transmission of vector-borne diseases and infections with invertebrate intermediate hosts is one area of health that is likely to be greatly affected by climate change, and changes in the distribution and seasonality of these infections and diseases may be among the first detectable changes in human health [4]. Indeed there is some evidence that there may have been climate-change driven changes in transmission already. Schistosomiasis transmission now occurs at altitudes above previously defined limits in Uganda [5,6], which may be due to higher temperatures. Many studies also suggest a link between increasing temperatures and the spread of malaria in eastern Africa and elsewhere [7–10], although others dispute this [11–13].

The effect of climate change on schistosomiasis has been largely neglected. In 1995, Martens *et al* [14] developed a simple population-based model of a generic snail and human schistosome population in relation to temperature, from which they concluded that increasing temperatures would expand the range of human schistosomiasis [14], but reduce transmission in areas where schistosomiasis is currently endemic [15]. In 2008 Zhou *et al* considered the minimum temperature requirements of *Oncomelania hupensis* and *Schistosoma japonicum* and used them to predict a northward shift in possible range for *S. japonicum* in China over the next few decades [16]. Most recently Mangal *et al* [17] simulated *S. mansoni* transmission at 20°C, 25°C, 30°C and 35°C using a non-species-specific *Biomphalaria* snail population and determined that the mean worm burden in humans increases between 20–30°C before falling at 35°C. The model also suggested that optimum control strategies may be different at different temperatures.

These models have a number of limitations. With the exception of the *O. hupensis* model [16], the models are parameterised using data from many different species of snail of the same genera [17], or even simulate generic snail and human schistosome populations [14,15]. They also do not take into account the large variation in cercaria production by temperature [14,15], or they do not include all temperature-sensitive stages of the schistosome lifecycle [17]. Different intermediate host snail species have very different habitat requirements and distributions, while snail experiments show that the relationship between temperature and mortality and recruitment rates varies between snail species [18]. We therefore simulate a specific snail species, *Biomphalaria pfeifferi*, which has a wide distribution in sub-Saharan Africa [19], and simulate all temperature-sensitive stages of the schistosome and snail lifecycles.

Methods

Model description

A dynamic, stochastic, agent-based model, written in NetLogo, was used to simulate the growth and mortality of a snail population, and infection by *S. mansoni*. The model has a time step of one hour. Each hour agents develop at temperature dependent rates; and can die, produce eggs, cause infection, etc with temperature dependent probabilities. The concept of 'heat units' is introduced in the model and used to track the development of snail eggs and juvenile snails, both of which take longer to reach maturity (hatch and become sexually mature respectively) away from optimum temperatures. Each heat unit is arbitrarily set to represent 1% of the total development needed to enter the next developmental stage. For instance, a juvenile snail living at a constant temperature of 15°C will take 32 hour to gain one heat unit, and 131 days to become sexually mature, and a juvenile snail living at 27°C will take 9 hours to gain one heat unit and 39 days to become mature. Figure 1 shows a diagram of the model structure. Full details of the model structure are in (Model description S1), and a brief description is given below.

Snails are born into the model as eggs. They accumulate heat units over time at a temperature dependent rate. When they accumulate 100 heat units they become juvenile snails [20]. A description of heat units is given in Model description S1. Like snail eggs, juvenile snails accumulate heat units at a temperature dependent rate over time, becoming adult snails when they have accumulated a further 100 heat units. Adult snails produce snail eggs at a rate that is dependent on temperature. Experimental studies suggest that extended periods of high temperatures during development are detrimental to reproductive development [21]. Egg production is therefore reduced in snails that were exposed to large numbers of degree hours above a threshold temperature as juveniles. Snail eggs, juveniles and adults die and are removed from the model at temperature dependent rates.

In laboratory conditions with a plentiful supply of food a high density of snails does not affect the time to first or maximum egg laying, or snail mortality rates [22]. It does however reduce the number of fertile eggs laid by each snail each week. Field studies support the idea that unfavourable conditions have a greater effect on egg production than snail mortality [23]. In the model, it is assumed that the environment can support 300 snails with no negative effect on egg production. Above this number, the rate of egg production drops. At population numbers of more than 600 snails, snail mortality rates also increase. Thresholds of 300 and 600 snails were chosen for practical reasons, with models with higher numbers taking longer to run, and models with smaller numbers requiring more runs in total to reduce stochasticity. With these thresholds, each model run takes approximately 3–5 minutes. These numbers can be scaled up or down with no effect on mean model results, provided that all parameters that are functions of snail population size, and the rate of miracidia introduction, are scaled accordingly.

Juvenile and adult snails in the model have one of three infection states: uninfected, prepatent or infectious. All snails are uninfected when they first hatch. Snails can change state from uninfected to prepatent and from prepatent to infectious only.

Miracidia are introduced into the model at a constant rate. No diurnal variation in the rate of introduction is simulated as schistosome eggs in stool hatch gradually over a period of many hours or days, as the stool breaks down in water [24]. This will have the effect of 'smoothing out' any diurnal variation in the rate of stool entering water bodies. Miracidia gain biological age at a temperature dependent rate, and die at a biological age dependent rate. An additional, temperature and age-independent mortality rate can also be simulated. Each hour, every miracidium in the model has the chance to infect a snail. The probability of infection is dependent on the biological age of the miracidium, the water temperature, and the number of snails in the model. When it is determined that a miracidium should infect a snail, the snail is chosen at random from all snails in the model. If the snail is uninfected, the snail becomes prepatent and the miracidium dies. If the snail is already prepatent or infectious then the miracidium dies, but there is no change to the snail.

Upon infection with a miracidium, uninfected juvenile and adult snails change infection state from uninfected to prepatent. They then start to gain heat units at a temperature dependent rate. When sufficient heat units have been accumulated, they change state to become infectious. Prepatent adult snails cease to produce eggs when they gain 50% of the heat units necessary to become infectious. Infectious snails do not produce eggs and have a higher mortality rate than prepatent and uninfected snails.

Infectious snails produce cercariae at rate which is dependent on temperature and the time of day. Like miracidia, cercariae gain biological age at a temperature dependent rate, and die at a biological age dependent rate. An additional, temperature and age-independent mortality rate can also be simulated.

The main output of our model is 'infection risk', a measure of the number of cercariae in the model adjusted by their decreasing probability of successfully causing infection with increasing biological age [25]. Human and adult worms are not simulated. This is because the worm stage of the parasite's lifecycle usually takes place inside a human host and is therefore unlikely to be affected by temperature. The link between human infection risk and snail infection risk is also unclear. It is likely that there is an overall positive correlation between cercaria numbers and

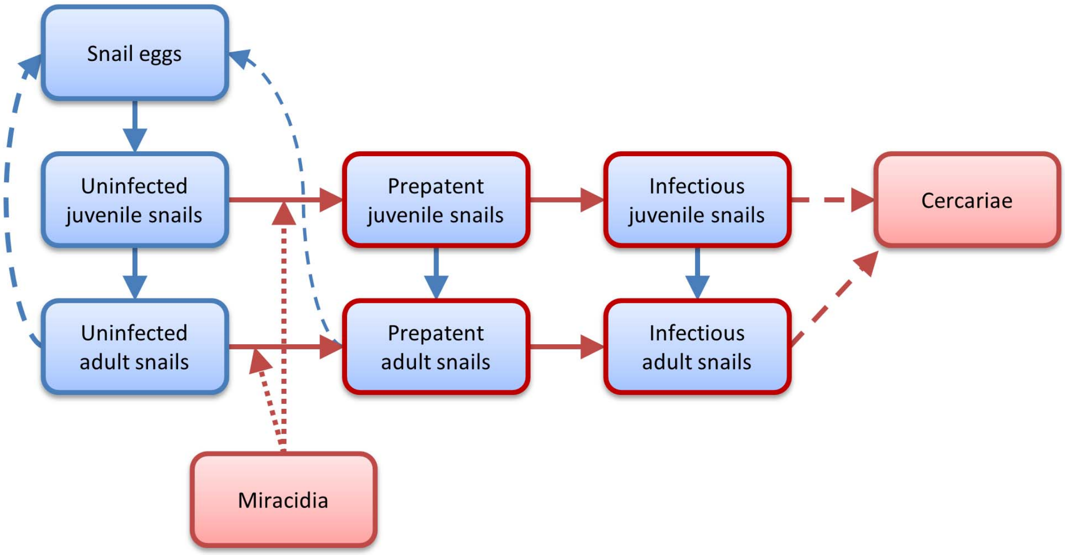

Figure 1. Diagram of the model structure. Boxes indicate classes of agents. Solid arrows indicate that agents can change from one class into another. Dashed lines indicate the production of one class by another. Dotted lines indicate infection. Red outlines and arrows indicate the presence of *Schistosoma mansoni*. Agents of all classes can die and be removed from the model. Table S1 contains details of the rates and probabilities that determine the movement of agents between classes.
doi:10.1371/journal.pone.0101462.g001

miracidium numbers, however differences in human water contact, defecation practices and migration will mean that the relationship varies greatly in different areas. Finally, the relationship between infection risk and the number of worms will depend on the overall prevalence and intensity of infection in an area (which will depend on human water contact and defecation practices as well cercariae numbers) as repeated infection leads to partial immunity [26]. For these reasons, humans and schistosome worms are not modelled explicitly. Instead, miracidia are introduced into the model at a constant rate and human infection risk is indicated by a function of the number of cercariae in the model and their probability of causing infection upon contact.

Data and model parameterisation

The model was parameterised using a combination of experimental and field data from *B. pfeifferi* and *S. mansoni*. All parameter values were based on empirical data. Full details are given in (Model description S1 and figures S1–S8 in Model description S1).

Simulated scenarios

Water temperatures were modelled as a sine wave. Three sets of scenarios with different levels of diurnal variation in temperature were modelled: one with constant temperatures, one where maximum and minimum temperatures varied from the mean temperature by 2°C, and one where they varied by 5°C. For each of the three sets of scenarios, two scenarios were modelled. In one, the 'lake' scenarios, cercariae and miracidia had temperature dependent mortality rates only, estimated from mortality rates in laboratory experiments. In the other, the 'river' scenarios, an additional temperature independent mortality rate of 0.5 per cercaria and miracidium per hour was simulated. The first scenario ('lake') approximates conditions in still water such as lakes and ponds and the second ('river') conditions in flowing water such as streams and rivers, where many miracidia and cercariae are likely to be quickly washed away. The scenarios are summarised in table 1. The lake and river scenarios can be thought of as extremes, with conditions in many water bodies falling somewhere between the two.

Each set of scenarios was run for all temperatures at which the simulated snail populations could survive indefinitely, with temperature increasing in 0.5°C increments.

The number of snails in the model is calculated as the total number of uninfected, prepatent and infectious juvenile and adult snails.

The model was run for five years to reach equilibrium (to become independent of initial conditions), and then outputs were averaged over two years and a minimum of 200 runs. Both mean daily infection risk and mean infection risk by hour of the day were calculated.

Results

Infection risk

In the lake scenario, human infection risk is highest at 15.0–19.0°C (figure 2). Compared to the lake scenario, infection risk in the river scenario is highest at higher temperatures, reaching its maximum at 20.5–25.0°C. In both the lake and river scenarios, with ±2°C and ±5°C diurnal variation in temperature there is a risk of infection at all temperatures at which snail populations can survive indefinitely. With no diurnal variation in temperature there is no infection risk below 15.0°C. In all lake scenarios, infection risk increases sharply as temperature increases above the minimum temperature at which transmission can occur. The increase in risk is more gradual in the river scenarios.

Both cercaria numbers and infection risk are highest between 3 pm and 5 pm in the lake scenario and between 1 pm and 3 pm in the river scenario (figure 3). In the lake scenario, infection risk remains high for longer at cooler temperatures. At 15.0°C, cercaria numbers are above 75% of their maximum numbers between 12 pm and 2 am and infection risk is above 75% of its maximum between 1 pm and 9–10 pm. At 29.5°C, cercaria numbers are above 75% of their maximum numbers between 1–2 pm and 8–9 pm and infection risk is above 75% of its maximum

Table 1. Summary of the simulated scenarios.

Lake ±0°C	Lake ±2°C	Lake ±5°C
- No additional temperature-independent cercaria and miracidium mortality rate- No diurnal variation in water temperature	- No additional temperature-independent cercaria and miracidium mortality rate- Daily minimum and maximum water temperatures are 2°C lower and higher than the mean water temperature	- No additional temperature-independent cercaria and miracidium mortality rate- Daily minimum and maximum water temperatures are 5°C lower and higher than the mean water temperature
**River ±0°C**	**River ±2°C**	**River ±5°C**
- An additional temperature-independent cercaria and miracidium mortality rate of 0.5 per cercaria/miracidium per hour is simulated- No diurnal variation in water temperature	- An additional temperature-independent cercaria and miracidium mortality rate of 0.5 per cercaria/miracidium per hour is simulated- Daily minimum and maximum water temperatures are 2°C lower and higher than the mean water temperature	- An additional temperature-independent cercaria and miracidium mortality rate of 0.5 per cercaria/ miracidium per hour is simulated- Daily minimum and maximum water temperatures are 5°C lower and higher than the mean water temperature

doi:10.1371/journal.pone.0101462.t001

between 1 pm and 5–6 pm. Times are given to the nearest hour as the model has a time-step of one hour. Compared to the lake scenario, in the river scenario both cercaria numbers and infection risk are high for shorter periods of time. Temperature has little effect on the relationship between time of day and cercariae numbers and infection risk in the river scenario, and both cercaria numbers and infection risk are above 75% of their maximum between 12 pm and 4 pm at all temperatures at which infection can occur. The amount of diurnal variation in temperature has little effect on the relationship between time of day and cercariae numbers and infection risk in all scenarios.

Snail population dynamics

The total number of snails in the model is approximately constant between 14.5–31.0°C when there is no or little (±2°C) diurnal variation in temperature, and between 15.5–29.0°C with a diurnal temperature range of ±5°C (figure 4). Either side of this snail numbers drop sharply, and the snail population cannot survive indefinitely outside the range 14.0–31.5°C at constant temperatures. Simulating a diurnal variation of ±2°C greatly increases snail population numbers at 14.0°C and decreases the highest temperature at which the snail population can survive indefinitely by 0.5°C. Simulating a diurnal variation of ±5°C reduces the range of mean temperatures within which the population can survive indefinitely to 15.0–29.5°C. There is no real difference between the snail populations in the lake and river scenarios (less than 2% difference in the total number of snails at the same temperature with the same amount of diurnal variation in temperature).

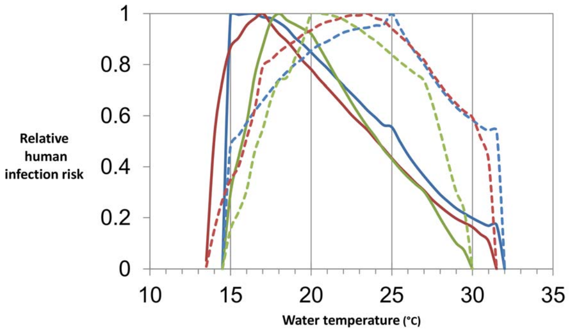

Figure 2. Relative human infection risk. The blues lines show risk with no diurnal variation in temperature, the red lines with ±2°C variation, and the greens lines with ±5°C variation. The solid lines show the results of the lake scenarios and the dashed lines the results of the river scenario. All risks are relative to maximum risk for the same scenario.
doi:10.1371/journal.pone.0101462.g002

Mean generation time, the time between an egg being laid and it first producing eggs, decreases with increasing constant temperature from 176 days at 14.0°C to a minimum of 46 days at 26.0°C before increasing slightly to 74 days at 32.0°C (figure 5). The number of days between a snail being infected and it first producing cercariae also decreases with increasing temperature, from 130 days at 14.0°C to 18 days at 32.0°C. The proportion of snails that are infectious decreases with increasing temperatures above 15.0–18.0°C in the lake scenario and 15.0–20.0°C in the river scenario, with the temperature at which the proportion of infectious snails peaks being highest with higher levels of diurnal variation in temperature (figure 6).

Discussion

We have developed a model that improves on earlier simulation work to greatly advance the field of schistosomiasis and climate change. We have done this by parameterising the model to a single species of snail, *B. pfeifferi*, which is involved in *S. mansoni* transmission throughout much of sub-Saharan Africa [19]. This paper, the first in a series, describes the model structure and parameterisation and looks in detail at the dynamics of the snail and parasite populations at different water temperatures. It considers the effects of diurnal temperature fluctuations and different types of water bodies on snail numbers and schistosomiasis transmission, and of time of day of water contact on infection risk. Future work, conducted as part of the HEALTHY FUTURES project [FP7 grant agreement no. 266327] (http://www.healthyfutures.eu/), will explore the effect of simulating different species of snail, and will run the model using climate change projections to obtain maps of future schistosomiasis risk in eastern Africa, relative to schistosomiasis risk today. This will also enable us to validate the model by comparing predicted risk maps produced by the model for the present day with empirical, geo-referenced data on schistosomiasis prevalence.

It is predicted that the global average surface temperature will be 1.1–6.4°C higher in 2090–2099, relative to 1980–1999 [27]. Our results suggest that in most lakes, ponds, reservoirs and dams where *B. pfeifferi* is an intermediate host for *S. mansoni* infection risk will decrease. In rivers and streams during seasons where mean temperatures are currently below around 20°C infection risk will increase. During seasons where mean temperatures are currently above around 25°C infection risk will decrease. In some

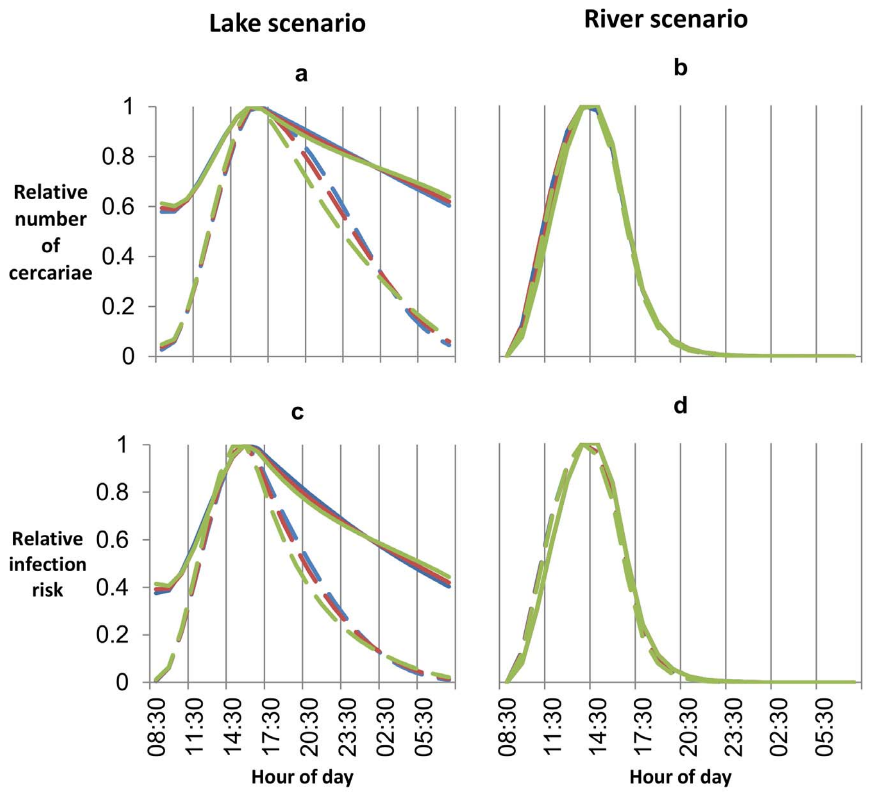

Figure 3. Relative cercariae numbers and relative infection risk by time of day in the lake and river scenarios. a) shows cercaria numbers in the lake scenario. b) shows cercaria numbers in the river scenario. c) shows infection risk in the lake scenario. d) shows infection risk in the river scenario. The blues lines show risk with no diurnal variation in temperature, the red lines with ±2°C variation, and the greens lines with ±5°C variation. Where the three colours cannot been seen the results are very similar regardless of levels of diurnal variation in temperature. The solid lines show results at a mean temperature of 15.0°C and the dashed lines at 29.5°C. Results are presented as cercaria numbers and infection risk, relative to maximum cercaria numbers and infection risk in the same scenario at the same temperature. Results for all temperatures between these two extremes lie between the lines for 15°C and 29.5°C, and are not shown.
doi:10.1371/journal.pone.0101462.g003

areas currently at the limits of *B. pfeifferi's* range snail populations may die out entirely.

Infection risk is highest between late morning and mid-afternoon in flowing water, and this does not vary with mean temperature. In still water bodies, infection risk remains high throughout the afternoon at high temperatures and into the evening at lower temperatures. An intriguing question posed by the model, and which will be explored further, is whether the ecological properties of a transmission site can or should be factored into control programmes. For example, behaviour change interventions aimed at reducing infection by encouraging people to avoid transmission sites at high risk times of day may be less feasible and effective at still water transmission sites, particularly where temperatures are low.

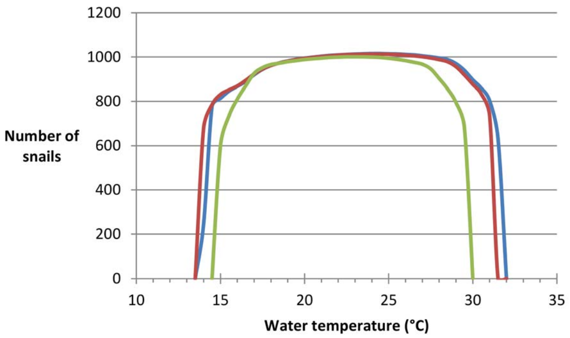

Figure 4. Number of snails by temperature. The blue line shows the total number of snails in the model with no diurnal variation in temperature, the red where the diurnal temperature varies by ±2°C, and the green where it varies by ±5°C. All are from the lake scenario, however results are very similar for the river scenario.
doi:10.1371/journal.pone.0101462.g004

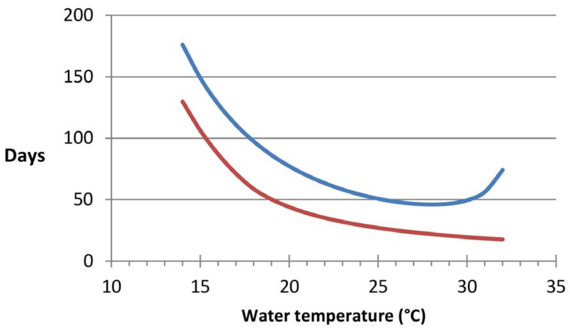

Figure 5. Snail generation time and prepatent period by temperature. The blue line shows the number of days between an egg being laid and it first producing eggs. The red line shows the number of days between a snail being infected and it first producing cercariae.
doi:10.1371/journal.pone.0101462.g005

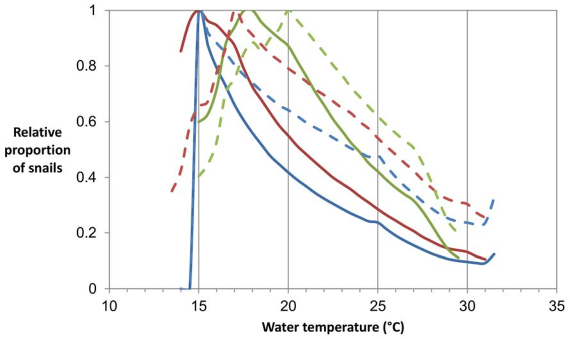

Figure 6. Proportion of snails that are infectious by temperature, relative to maximum proportion in the same scenario. The blues lines show risk with no diurnal variation in temperature, the red lines with ±2°C variation, and the green lines with ±5°C variation. The solid lines show the results of the lake scenarios and the dashed lines the results of the river scenario.
doi:10.1371/journal.pone.0101462.g006

Snail generation times will decrease as temperatures increase as eggs take less time to hatch at higher temperatures, and juvenile snails take less time to start producing eggs. This means that snail populations will recover faster from seasonal reductions in their numbers. Snail populations will also take less time to regain their original numbers following any snail control efforts, meaning that intervals between molluscicide applications and other control methods will need to be reduced to have the same effect. In addition, the length of time between a snail being infected and it first producing cercariae decreases with increasing temperature (figure 3), meaning that infection risk will also take less time to regain its pre-control levels.

Our findings show that the proportion of snails that are infectious at a transmission site cannot be used as a reliable measure of relative infection risk without water temperature being taken into consideration. At sites with lower temperatures, all else being equal, a higher proportion of snails will be infectious, but this does not necessarily translate into a higher risk of infection. This is largely due to the fact that cercaria production by each infected snail increases greatly with increasing temperature (figure S9). The number of cercariae also does not correspond directly with infection risk, as cercariae become less infectious with age. Finally, changes in temperature may lead to changes in infection risk, without having a large effect on total snail population numbers.

In our model there is a small range of temperatures and conditions at which *B. pfeifferi* populations can survive but at which little or no sustained transmission of *S. mansoni* can occur. This is in line with empirical data, with studies reporting the existence of *B. pfeifferi* or other *Biomphalaria* populations at low temperatures with no or only seasonal schistosomiasis transmission occurring [28–30]. This band of temperatures is relatively narrow however, and it is therefore probable that the majority of cooler areas where *B. pfeifferi* snails are currently found will become suitable for schistosomiasis transmission over coming decades.

We present results for two scenarios with different levels of cercaria and miracidium mortality: 'lake' and 'river'. The scenario 'lake' is designed to represent conditions where the majority of cercaria and miracidia mortality occurs as a result of depletion of glycogen stores. The scenario 'river' represents conditions where the rate of cercaria and miracidium mortality or local depletion is high. In general conditions in still water bodies will be best approximated by the 'lake' scenario and conditions in flowing water by the 'river' scenario, but there will be many exceptions. For example, in lakes where predation of cercariae and miracidia is high the 'river' scenario may be more appropriate, and in pockets of calm water at the edge of rivers local conditions may be better approximated by the 'lake' scenario. Furthermore, the conditions in many water bodies may fall somewhere between the two scenarios.

Our model does not simulate the link between cercaria numbers and miracidium numbers. While this means that the model does not make assumptions about the relationship between the two, which will be different in different populations and is unlikely to be simply linear, it also means that there is no feedback between cercaria numbers and miracidium numbers. Although this will have impacted on absolute infection risk in the model, it is unlikely to have had a large effect on the overall relationship between temperature and relative infection risk. It may however mean that the lower temperature bounds for schistosomiasis transmission estimated by our model are too low in some settings. In particular, in settings where human risk behaviour is low (good sanitation and/or little water contact) and where human migration from areas with more intense schistosomiasis transmission is low.

There are a number of issues that need to be considered when using our model to predict the effects of climate change on schistosomiasis transmission. The first is that the temperatures in our model are the water temperatures experienced by the snails and parasite and will not correspond directly with air temperature for a number of reasons [20]. In particular, surface water temperatures tend to be slightly higher than air temperatures and snails in large water bodies can often avoid above-optimum temperatures by moving to deeper water or burrowing in mud. Second, our model investigates the effect of changes in temperature only. Changes in seasonality and the size, permanency and flow rate of water bodies may also result from climate change, and will also lead to changes to schistosomiasis transmission [20,31]. Previous studies have shown that other environmental factors such as water conductivity may also influence snail numbers [32]. These environmental factors may change as a result of climate change. In addition, increasing temperatures will also have an effect on both the snails' food sources and their predators, complicating the relationship between increasing temperature and snail numbers. Finally, while *B. pfeifferi* is the most widespread and most important intermediate host for *S. mansoni* in sub-Saharan Africa [18,19], other snail species are responsible for transmission in some areas. We believe that the overall findings of this study are likely to apply to areas with other snail hosts, but the minimum and maximum temperatures for snail and parasite survival and the temperatures at which infection risk is highest may vary [33].

Our results suggest that increasing temperatures will increase schistosomiasis risk in flowing water in cooler areas and decrease it in warmer areas and in still water bodies. They also suggest that areas where *B. pfeifferi* snails are currently found but where little or no transmission occurs will become suitable for transmission of schistosomiasis over coming years and decades. Furthermore, infection risk increases sharply once the minimum temperature necessary for transmission is reached, particularly in still water bodies, meaning that once the parasite is introduced into these areas epidemics of schistosomiasis could occur. This is of particular concern as many of these areas will fall outside current control programs and people at risk may have little knowledge of or immunity to schistosomiasis. There is therefore an urgent need for these areas to be monitored to minimise the impact of future

epidemics and to keep schistosomiasis control and elimination plans on track.

Supporting Information

Model Description S1 Additional information on the model structure and parameterisation.
(DOCX)

Acknowledgments

The authors would like to thank Healthy Futures project partners who commented on a draft of the manuscript, and Marc Savy for writing an averaging script.

Author Contributions

Conceived and designed the experiments: NM. Performed the experiments: NM. Analyzed the data: NM. Contributed to the writing of the manuscript: NM. Reviewed and revised the manuscript: MB.

References

1. IPCC (1990) Climate Change: The IPCC Impacts Assessment. Australian Government Publishing Service, Camberra, Australia.
2. IPCC (2007) Climate Change 2007: Impacts, Adaptation and Vulnerability. Contribution of Working Group II to the Fourth Assessment Report of the Intergovernmental Panel on Climate Change. Cambridge University Press, Cambridge, UK.
3. World Health Assembly (2001) Schistosomiasis and soil-transmitted helminth infections. Resolution WHA54.19. Geneva.
4. World Health Organization (2003) Climate Change and Human Health - Risks and Responses. Geneva.
5. Kabatereine NB, Brooker S, Tukahebwa EM, Kazibwe F, Onapa AW (2004) Epidemiology and geography of Schistosoma mansoni in Uganda: implications for planning control. Trop Med Int Health 9: 372–380.
6. Rubaihayo J, Moghusu E, Clouds P, Abaasa A (2008) Schistosomiasis transmission at high altitude crater lakes in Western Uganda. BMC Infectious Diseases 8: 110.
7. Pascual M, Ahumada JA, Chaves LF, Rodó X, Bouma M (2006) Malaria resurgence in the East African highlands: Temperature trends revisited. Proceedings of the National Academy of Sciences 103: 5829–5834.
8. Patz JA, Hulme M, Rosenzweig C, Mitchell TD, Goldberg RA, et al. (2002) Climate change (Communication arising): Regional warming and malaria resurgence. Nature 420: 627–628.
9. Cohen J, Smith D, Cotter C, Ward A, Yamey G, et al. (2012) Malaria resurgence: a systematic review and assessment of its causes. Malaria Journal 11: 122.
10. Chaves LF, Koenraadt CJ (2010) Climate change and highland malaria: fresh air for a hot debate. Q Rev Biol 85: 27–55.
11. Hay SI, Cox J, Rogers DJ, Randolph SE, Stern DI, et al. (2002) Climate change and the resurgence of malaria in the East African highlands. Nature 415: 905–909.
12. Hay SI, Rogers DJ, Randolph SE, Stern DI, Cox J, et al. (2002) Hot topic or hot air? Climate change and malaria resurgence in East African highlands. Trends in Parasitology 18: 530–534.
13. Mouchet J, Manguin S, Sircoulon J, Laventure S, Faye O, et al. (1998) Evolution of malaria in Africa for the past 40 years: impact of climatic and human factors. J Am Mosq Control Assoc 14: 121–130.
14. Martens WJM, Jetten TH, Rotmans J, Niessen LW (1995) Climate change and vector-borne diseases: A global modelling perspective. Global Environmental Change 5: 195–209.
15. Martens WJM, Jetten TH, Focks DA (1997) Sensitivity of malaria, schistosomiasis and dengue to global warming. Climatic Change 35: 145–156.
16. Zhou XN, Yang GJ, Yang K, Wang XH, Hong QB, et al. (2008) Potential impact of climate change on schistosomiasis transmission in China. Am J Trop Med Hyg 78: 188–194.
17. Mangal TD, Paterson S, Fenton A (2008) Predicting the impact of long-term temperature changes on the epidemiology and control of schistosomiasis: a mechanistic model. PLoS ONE 3: e1438.
18. Brown D (1994) Freshwater Snails Of Africa And Their Medical Importance. London, UK: Taylor & Francis.
19. Stensgaard A-S, Utzinger J, Vounatsou P, Hürlimann E, Schur N, et al. (2013) Large-scale determinants of intestinal schistosomiasis and intermediate host snail distribution across Africa: Does climate matter? Acta Tropica 128: 378–390.
20. McCreesh N, Booth M (2013) Challenges in predicting the effects of climate change on Schistosoma mansoni and Schistosoma haematobium transmission potential. Trends in Parasitology 29: 548–555.
21. Appleton CC, Eriksson IM (1984) The influence of fluctuating above-optimal temperature regimes on the fecundity of Biomphalaria pfeifferi (Mollusca: Planorbidae). Transactions of the Royal Society of Tropical Medicine and Hygiene 78: 49–54.
22. Loreau M, Baluku B (1987) Growth and demography of populations of *Biomphalaria pfeifferi* (gastropoda, planorbidae) in the laboratory. Journal of Molluscan Studies 53: 171–177.
23. Loreau M, Baluku B (1987) Popoulation dynamics of the freshwater snail Biomphalaria pfeifferi in Eastern Zaire. Journal of Molluscan Studies 53: 249–265.
24. Upatham ES (1972) Rapidity and duration of hatching of St. Lucian Schistosoma mansoni eggs in outdoor habitats. J Helminthol 46: 271–276.
25. Ghandour AM, Webbe G (1973) A study of the death of Schistosoma mansoni cercariae during penetration of mammalian host skin: the influence of the ages of the cercariae and of the host. Int J Parasitol 3: 789–794.
26. Pinot de Moira A, Fulford AJC, Kabatereine NB, Ouma JH, Booth M, et al. (2010) Analysis of Complex Patterns of Human Exposure and Immunity to Schistosomiasis mansoni: The Influence of Age, Sex, Ethnicity and IgE. PLoS Negl Trop Dis 4: e820.
27. IPCC (2007) Summary for Policymakers. Cambridge, United Kingdom and New York, NY, USA.
28. Kloos H, Lo C, Birrier H, Ayele T, Tedla S, et al. (1988) Schistosomiasis in Ethiopia. Social Science and Medicine 26: 803–827.
29. Shiff CJ, Evans A, Yiannakis C, Eardley M (1975) Seasonal influence on the production of Schistosoma haemotobium and S. mansoni cercariae in Rhodesia. Int J Parasitol 5: 119–123.
30. Chu KY, Dawood IK (1970) Cercarial transmission seasons of Schistosoma mansoni in the Nile Delta area. Bull World Health Organ 42: 575–580.
31. Kariuki HC, Madsen H, Ouma JH, Butterworth AE, Dunne DW, et al. (2013) Long term study on the effect of mollusciciding with niclosamide in stream habitats on the transmission of schistosomiasis mansoni after community-based chemotherapy in Makueni District, Kenya. Parasit Vectors 6: 107.
32. Kazibwe F, Makanga B, Rubaire-Akiiki C, Ouma J, Kariuki C, et al. (2006) Ecology of Biomphalaria (Gastropoda: Planorbidae) in Lake Albert, Western Uganda: snail distributions, infection with schistosomes and temporal associations with environmental dynamics. Hydrobiologia 568: 433–444.
33. McCreesh N, Booth M (2014) The effect of simulating different intermediate host snail species on the link between water temperature and schistosomiasis risk. PLOS ONE.
